# Modelling malaria in southernmost provinces of Thailand: a two-step process for analysis of highly right-skewed data with a large proportion of zeros

**DOI:** 10.1186/s12936-022-04363-8

**Published:** 2022-11-15

**Authors:** Lumpoo Ammatawiyanon, Phattrawan Tongkumchum, Apiradee Lim, Don McNeil

**Affiliations:** Department of Mathematics and Computer Science, Faculty of Science and Technology, Prince of Songkla University, Pattani Campus, Pattani, 94000 Thailand

**Keywords:** Malaria, Two-step process, Southernmost provinces of Thailand

## Abstract

**Background:**

Malaria remains a serious health problem in the southern border provinces of Thailand. The issue areas can be identified using an appropriate statistical model. This study aimed to investigate malaria for its spatial occurrence and incidence rate in the southernmost provinces of Thailand.

**Methods:**

The Thai Office of Disease Prevention and Control, Ministry of Public Health, provided total hospital admissions of malaria cases from 2008 to 2020, which were classified by age, gender, and sub-district of residence. Sixty-two sub-districts were excluded since they had no malaria cases. A logistic model was used to identify spatial occurrence patterns of malaria, and a log-linear regression model was employed to model the incidence rate after eliminating records with zero cases.

**Results:**

The overall occurrence rate was 9.8% and the overall median incidence rate was 4.3 cases per 1,000 population. Malaria occurence peaked at young adults aged 20–29, and subsequently fell with age for both sexes, whereas incidence rate increased with age for both sexes. Malaria occurrence and incidence rates fluctuated; they appeared to be on the decline. The area with the highest malaria occurrence and incidence rate was remarkably similar to the area with the highest number of malaria cases, which were mostly in Yala province's sub-districts bordering Malaysia.

**Conclusions:**

Malaria is a serious problem in forest-covered border areas. The correct policies and strategies should be concentrated in these areas, in order to address this condition.

## Background

Malaria has been a plague on humanity since antiquity and continues to be so now**.** It is caused by *Plasmodium* protozoan parasites and spread by *Anopheles* mosquitoes [1]**.** In the 87 developing countries, about half of the world’s population lives in high-risk malaria transmission zones, especially in tropical and subtropical rural areas [2]**.** There were an estimated 241 million malaria cases in 2020, with 627,000 deaths worldwide [3]**.** Southeast Asia region contributes about 10**%** and 2**%** of global new cases and deaths, respectively, in 2020, making it the second-largest contributor region to the global malaria burden [3]**.**

In Thailand, the occurrence and transmission of malaria remain high along the international borders with Cambodia, Myanmar, and Malaysia [4–8]. There is significant geographical heterogeneity in the spatial distribution of malaria incidence, with some regions having little or no incidence of malaria, while other regions remain endemic, especially the rural forest and forest fringe areas [9]. The majority of cases were people who worked in forests, orchards, rubber plantations, and farms [10, 11]. Over 13 million people in Thailand (19% of the total population) are currently at risk for malaria and more than 200,000 live in focal active malaria areas [3].

Thailand has established the goal of eradicating malaria by 2024 and recently announced malaria-free status in 42 of its 77 provinces [12]. Despite this achievement, malaria continues to be concentrated along Thailand’s borders, making the effort to eradicate the illness much more difficult. The northeast, bordering Lao and Cambodia (especially Ubon Ratchathani and Sisaket provinces), the west, bordering Myanmar (particularly Tak province), and the south, bordering Malaysia (specifically Yala province) are the three main hotspots of malaria transmission in Thailand [9, 13–15]. The movement of migrant workers who may travel from malaria-endemic areas, a lack of access to malaria prevention, diagnostic and treatment options, and inadequate monitoring measures could all contribute to malaria outbreaks in border regions [9, 16, 17]. The border area is typically densely forested, which serves as a breeding ground for malaria vectors. Due to the remoteness of these places, malaria control might be challenging.

The southernmost provinces of Thailand have long experienced political and social unrest that has hampered malaria control activities [9, 18]. The four southernmost provinces, namely Songkhla, Pattani, Yala, and Narathiwat, cover a total land area of 18,330 square kilometres with a population of over 4 million in 2021 [19]. These provinces are located approximately 1000 km south of Bangkok. The Songkhla, Yala, and Narathiwat provinces share a border with Malaysia at various points. The climate in the southernmost provinces of Thailand is characterized as tropical. The average annual temperature in this area is 27.7 degrees Celsius (°C), with average annual minimum and maximum temperatures of 24 °C and 32.3 °C, respectively [20]. Also, the area has mountain ranges and rainforest jungles. The forests and mountains present a breeding ground for mosquitoes and other disease-transmitting insects. This area has consistently been among the provinces with the highest malaria morbidity in Thailand [9]. Malaria incidence has primarily been studied on Thailand's western and eastern borders with Myanmar and Cambodia, while study on malaria in Thailand’s southern border region with Malaysia is still scarce.

The number of malaria cases in Thailand has decreased [21] and the distribution of cases at sub-district levels is becoming increasingly sporadic as areas progress towards elimination especially southernmost provinces of Thailand [22]. As a result, there were no cases in many sub-districts. The excess of zero cases is an analytical challenge. Excessive zeros commonly occur in many application fields of statistics, including ecology, environmental science, biostatistical, and epidemiological study. The high proportion of zeros can lead to overdispersion, and this means a disagreement between the data and the assumed distribution. In other words, the malaria data generally had more zeros than the proposed distribution could reasonably explain. The zero-valued data should not be removed from the analysis. In addition, having a large proportion of zeros could indicate an important condition under study. Therefore, this study aimed to identify the incidence of malaria in each sub-district adjusted for gender, age group, and year by using a two-step method for analysing highly right-skewed data distributions with a high fraction of zeros.

## Methods

### Study areas

**Fig. 1:**
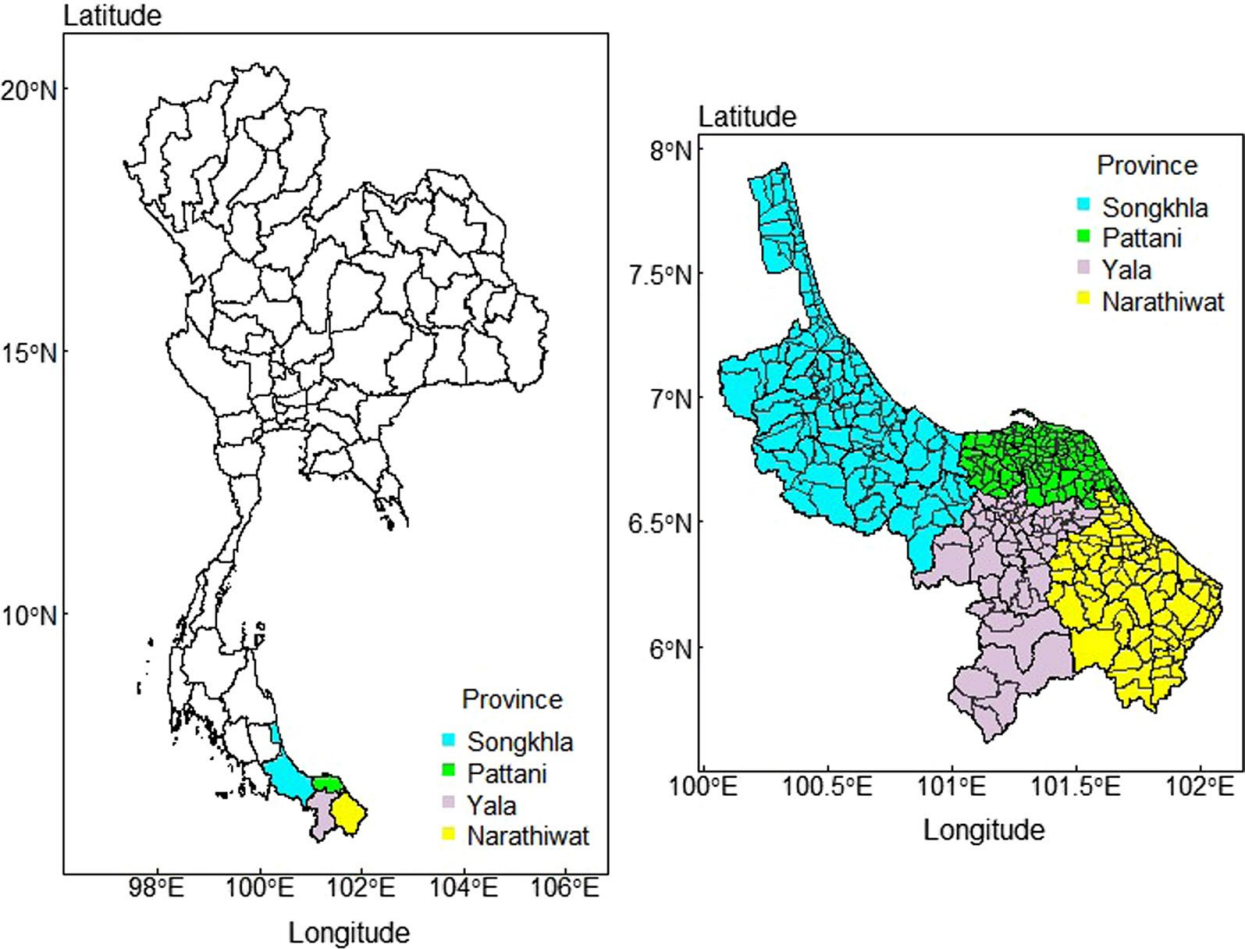
The study area comprises the four southernmost provinces of Thailand

The study areas covered the four southernmost provinces of Thailand including Songkhla, Pattani, Yala and Narathiwat. These four provinces consist of 49 administrative districts and 377 sub-districts as shown in Fig. 1.

### Data sources and management

In Thailand, there are two sources of malaria data: hospital admissions (reported to the Bureau of Epidemiology by the General Health Services) and malaria clinic and other facility treatment (reported to the Division of Vector Borne Diseases). In this study, hospital admission data were the only source of malaria data. The hospitalizations were diagnosed by physicians and some cases were confirmed by rapid diagnostic tests or blood smears for malaria. The tests were administered in all community hospitals, and the results were entered into a database.

The hospital admission data for malaria from four southernmost provinces, classified by age, gender, date of admission, sub-district of residence and citizenship were obtained from the Office of Disease Prevention and Control, Ministry of Public Health. Number of population in year 2008–2020 by age, gender, sub-district and year were downloaded from the website of the Department of Provincial Administration in Thailand’s Ministry of Interior.

Total hospital admissions and populations of sub-districts in 2008–2020 were used for data analysis. The incidence rates were computed by dividing the numbers of disease cases by the corresponding populations at risk.

Gender and age were grouped together into 16 levels (with eight levels of age in years: 0–19, 10–19, 20–29, 30–39, 40–49, 50–59, 60–69, and 70+) and named gender-age group. There were 377 sub-districts (with 127 sub-districts of Sonkhla, 115 sub-districts of Pattani, 58 sub-districts of Yala, and 77 sub-districts of Narathiwat). The total number of years was thirteen. Throughout a 13-year period, there were no cases in 62 sub-districts, therefore they were excluded from this study.

### Statistical models

In many cases, the Poisson model for disease counts fails due to excess variation in the data, in which case biostatisticians prefer to fit a negative binomial model with an over-dispersion parameter, θ, where smaller values of θ correspond to greater dispersion. In this case, the fit of a negative binomial model with a very small value of θ is still poor when there are many zeros in the data, in which case biostatisticians use zero-inflated or hurdle models.

In this study, an alternative model was purposed. The model simplifies fitting the zero-inflated model by separating into occurrence and incidence rate. This allows separate models to be fitted to the data for these two outcomes, which could have different predictor patterns. An occurrence is coded as 1 if the record contains at least one positive outcome, and 0 otherwise. There were 65,520 records (comprising 16 gender-age groups, 13 years, and 315 sub-districts) were used for analysing occurrence within each year, gender, age group, and sub-district.

An occurrence was modelled simply using logistic regression. Gender-age group, year and sub-district were put into the model as predictors. The population by gender-age group, year and sub-district was divided into four groups: less than 400, 400–599, 600–799 and 800 or more persons and it was included as an extra determinant. The population group was included because it is possible that records with relatively large populations are more likely to have an occurrence. The model was fitted using the following equation:

$$\mathit{ln}\left(\frac{{p}_{ijkl}}{1-{p}_{ijkl}}\right)=\mu +{\alpha }_{i}+{\beta }_{j}+{\delta }_{k}+{\gamma }_{l}$$

The term ${p}_{ijkl}$ denotes the outcome probability in a combination of predictive factor levels. The terms ${\alpha }_{i}$, ${\beta }_{j}$, ${\delta }_{k}$ and ${\gamma }_{l}$ thus represent effects of gender-age group, year, sub-district and population group. In this model, the outcome probability is expressed as the following equation:

$${p}_{ijkl}=\frac{1}{1+\mathit{exp}\{-(\mu +{\alpha }_{i}+{\beta }_{j}+{\delta }_{k}+{\gamma }_{l})\}}$$

The incidence rate is the number of cases divided by the population, given that there is at least one.

There were 59,087 records without cases and 6,433 records with cases. Only the records with non-zero cases were used to compute the incidence rate. A log-linear regression model can be used to model the incidence rate, which uses logarithms of incidence rates to make the data have a normal distribution. The log-linear model was fitted using the following equation:

$$\mathrm{ln}\left(\frac{{n}_{ijk}}{{P}_{i}}\right)={y}_{ijk}=\mu +{\alpha }_{i}+{\beta }_{j}+{\delta }_{k}$$

In this model, ${P}_{i}$ is the population in a sub-district, and ${n}_{ijk}$ is the corresponding number of reported cases in sub-district *k* and gender-age group *i* of the year *j*.

Instead of using the first level of the model as the reference, as is the case with traditional treatment contrasts, sum contrasts were used [23]. This method allows for the computation of an estimate and the 95% confidence interval of the occurrence and incidence rates for levels of each predictive factor in the models. A confidence interval plot was used to divide levels of a predictor into three groups, depending on the placement of these intervals completely above, around, or below a specified level. The thematic map was created by classifying sub-districts according to whether their malaria occurrence is above or below the overall mean, while another thematic map was created by classifying sub-districts according to whether their malaria incidence rate is above or below the overall median.

To assess the accuracy of model prediction, the Receiver Operating Characteristic (ROC) curve from logistic regression was drawn. The area under the ROC curve (AUC) measures the performance of a model and represents model accuracy. Linear regression models assume that errors are normally distributed, and this assumption is best assessed by a quantile–quantile (Q–Q) plot of studentised residuals.

Results from the models are shown as confidence interval plots and thematic maps. All statistical analysis and graphical displays were done using the R program version 3.4.4 [24].

## Results

**Fig. 2:**
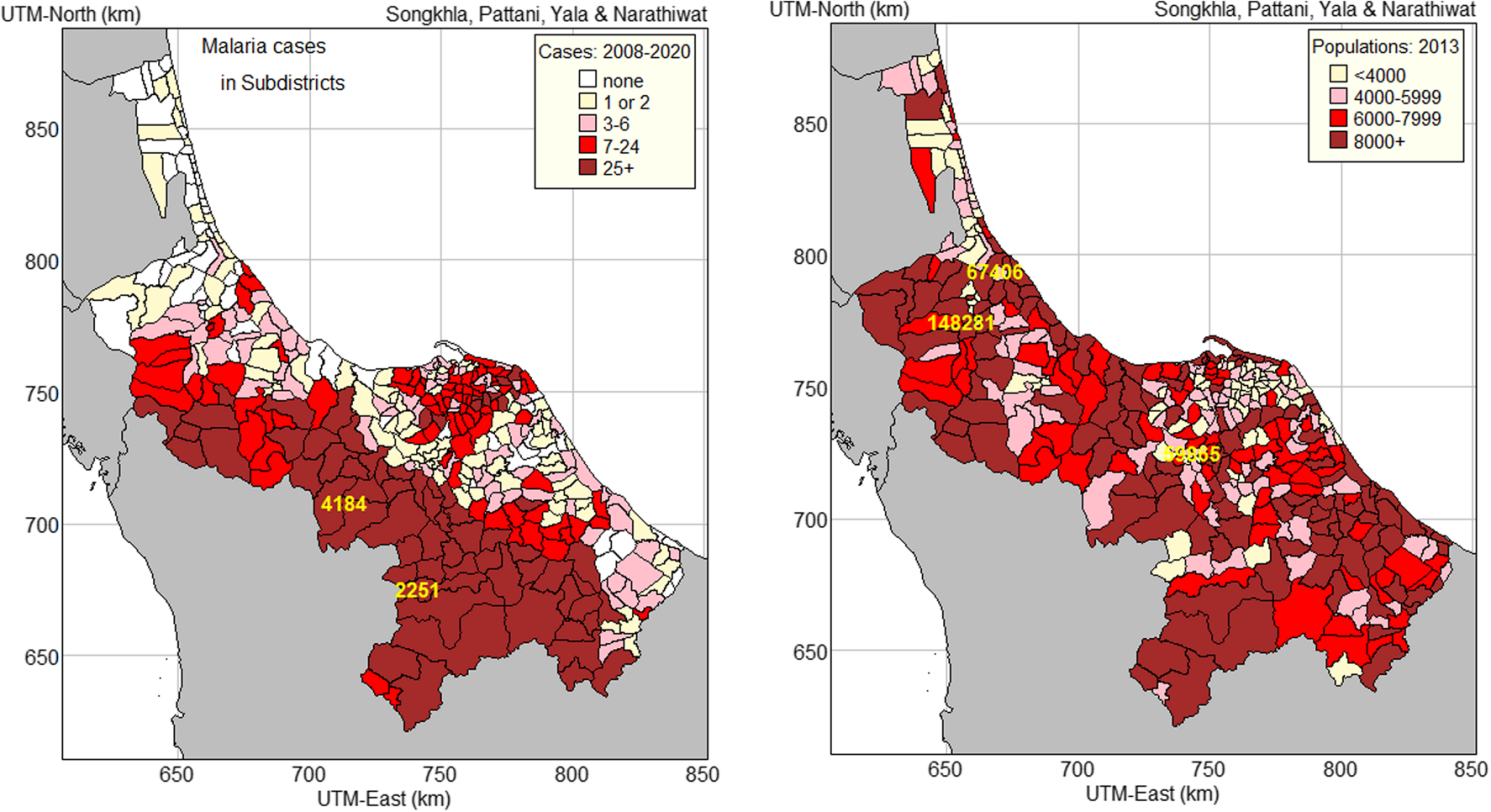
The number of malaria cases in 2008–2020 and the population count in 2013, shown by sub-district in the four southernmost provinces of Thailand

Total hospital admissions between 2008 and 2020 ranged from 0 (62 sub-districts) to 4184 times (Balah sub-district in Yala), with high numbers in mountainous areas along the southern border with Malaysia (Fig. 2, left map). The 2013 populations of sub-districts in all four provinces were chosen to illustrate in the right map of Fig. 2. The population ranged from 1678 (Ta Che sub-district in Yala) to 148,281 (Hat Yai sub-district in Songkhla).

The 62 sub-districts with no cases for a consecutive 13-year period were omitted. Only 6433 records out of 65,520 in the study region had a malaria occurrence, resulting in a 9.8% occurrence rate. The disease incidence rate is defined as the corresponding incidence rate per 1,000 population.

**Fig. 3:**
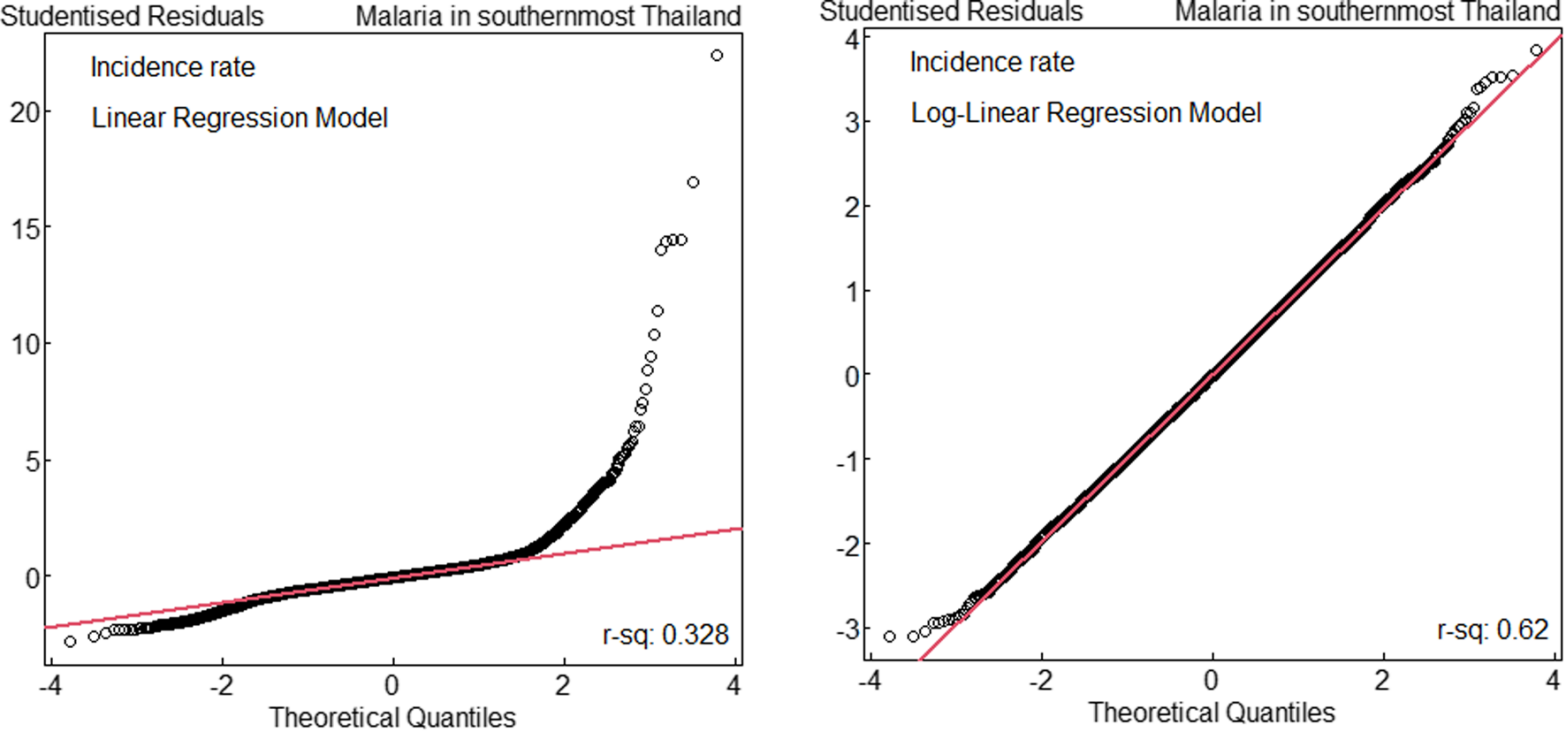
Quantile–quantile (Q–Q) plots of studentised residuals from linear and log-linear models

A linear model for predicting the malaria incidence using gender-age group, year, and sub-district as predictive factors gives a very poor fit, as shown by the Q–Q plots of the studentised residuals. This is because malaria incidence has a highly right-skewed distribution. The model fits quite well and the R^2^ nearly doubles from 32.8 to 62.0% when we fit the same linear model to the logarithm of incidence rates (Fig. 3).

**Fig. 4:**
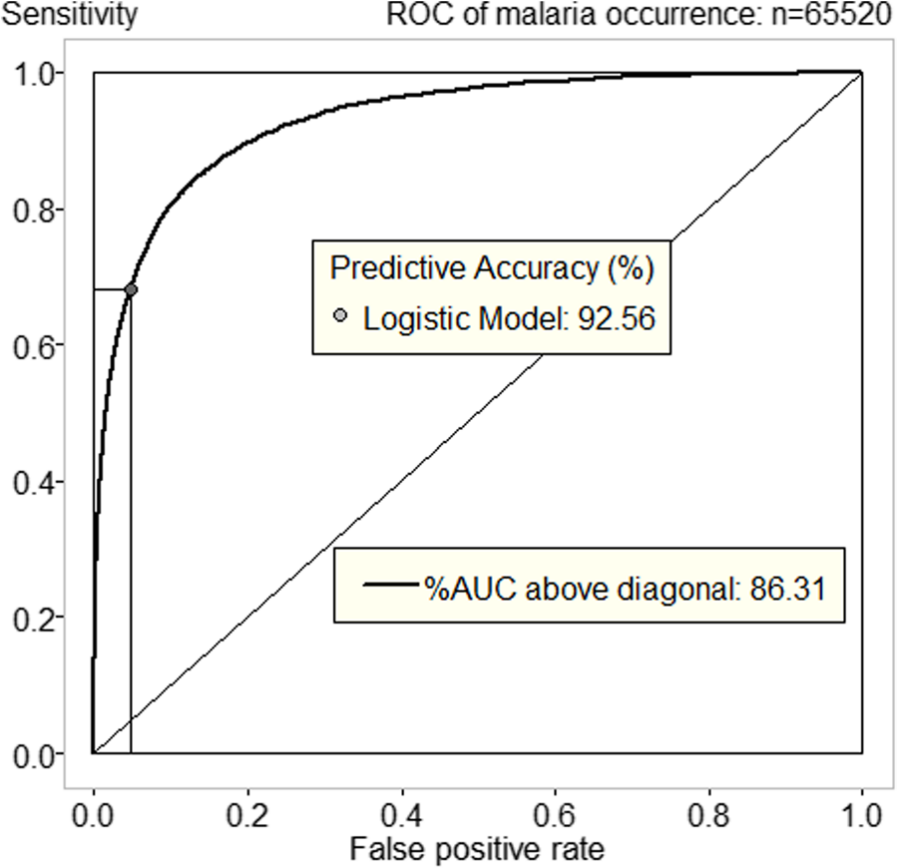
ROC curve from the logistic regression model

A logistic model for predicting the malaria occurrence using gender-age group, year, sub-district, and population group as predictive factors was assessed using ROC curve. The ROC curve shows how well a model predicts a binary outcome. It plots sensitivity (probability of finding an outcome when it is there) against the false positive error rate (probability of finding an outcome when it is not there). The cut-off point marked by the dot gives a total predicted number agreement of the number of records in the model. The ROC curve demonstrates that a model including gender-age group, year, sub-district and population group fits the occurrence data very well with an AUC of 0.8631 (Fig. 4), and it provides 92.56% predicted accuracy.

**Fig. 5:**
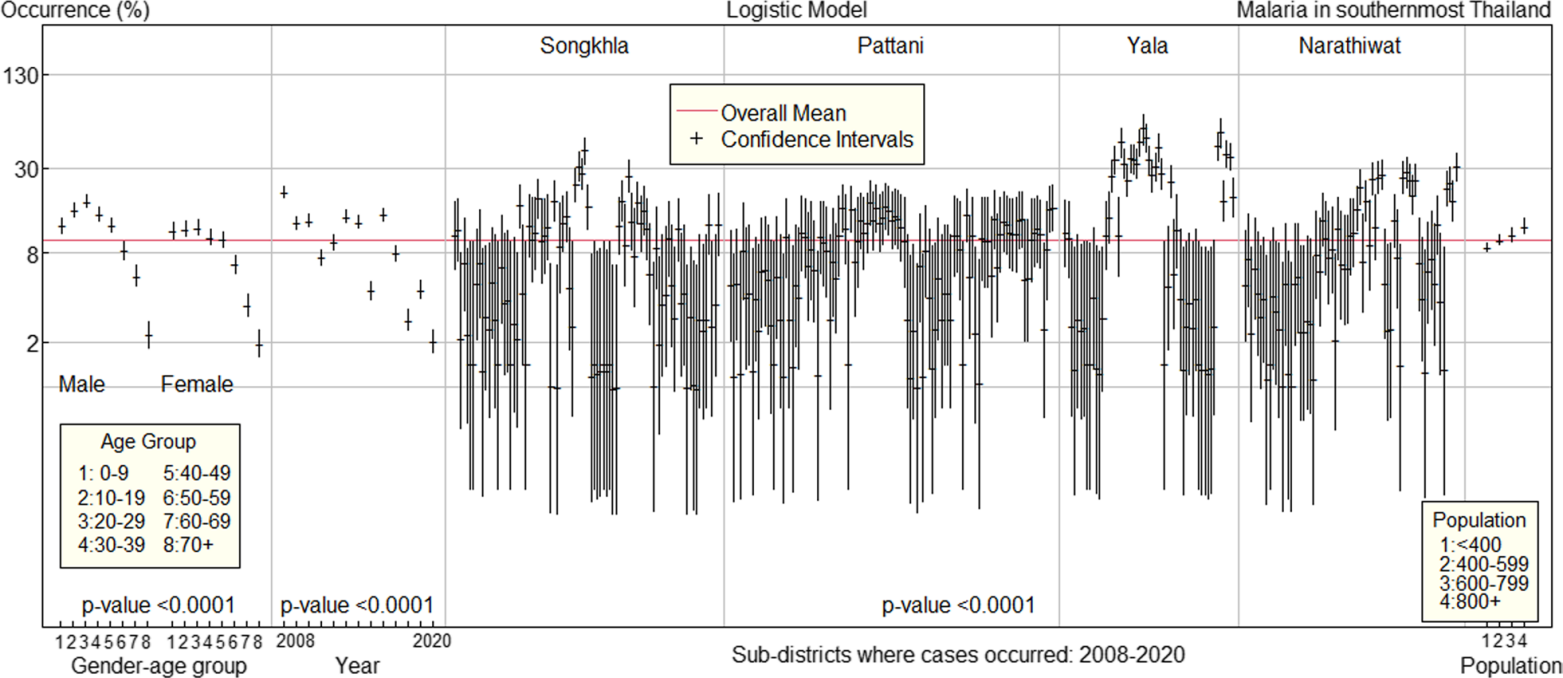
Malaria occurrence in 2008–2020 for each predictive factor in four southernmost provinces of Thailand

Confidence intervals of malaria occurrence for levels of each predictive factor from the logistic regression model were plotted with an overall mean of 9.8% (Fig. 5). Malaria occurrence peaked in males between the ages of 20 and 29, while it peaked in females at a younger, wider age and then declined as age increased in both genders. Sub-districts varied, with areas of high occurrence, particularly the majority of sub-districts in Yala province, moderate sub-districts in Narathiwat province, and a few sub-districts in Songkhla province. Less variation was evident in the sub-districts of the Pattani province. Malaria occurrence increased as population increased.

**Fig. 6:**
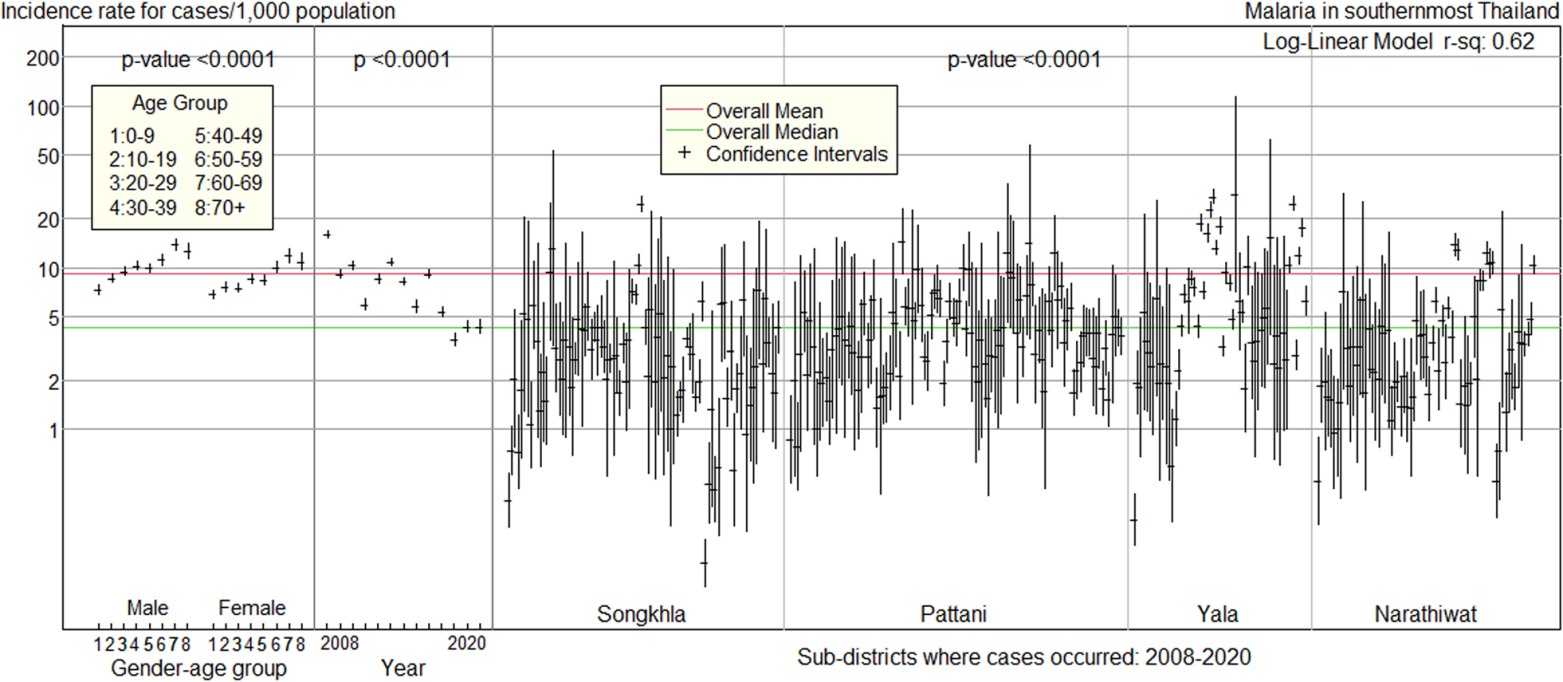
Incidence rate of malaria in 2008–2020 for each predictive factor in southernmost Thailand

The log-linear model's confidence intervals for the malaria incidence rate for each predictive factor were plotted **(**Fig. 6**)**. The overall median incidence rate was 4.3 cases per 1000 population, whereas the overall mean was 9.2 cases per 1000 population. The larger value of the overall mean than the overall median is caused by a highly right-skewed distribution of malaria incidence rate. This does not affect the results from the model because the model was fitted to logarithms of incidence rates, which are normally distributed.

The incidence rate patterns showed moderate increases with age for each gender, a decline over the decade from 2008 to 2020, and high variation among sub-districts, with pockets of higher incidence rates than the average in Yala and Narathiwat provinces and one sub-district in Songkhla province.

**Fig. 7:**
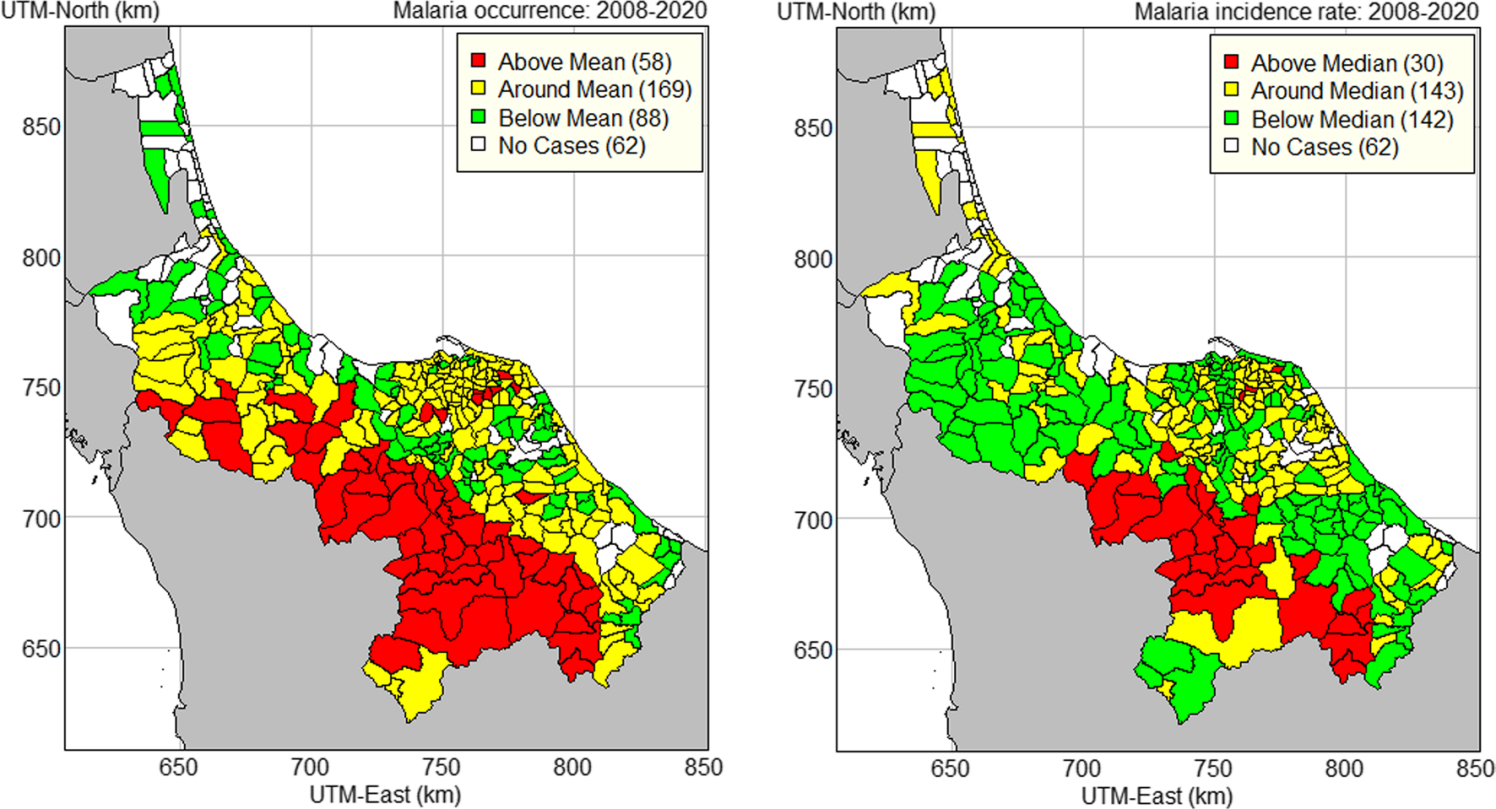
Thematic maps of malaria occurrence and malaria incidence rate in 2008–2020 by sub-district in the four southernmost provinces of Thailand

A confidence interval plot of malaria incidence rate was used to divide sub-districts into three groups, depending on the placement of these intervals completely above, around, or below the overall median (Fig. 7, right map). The red colour indicates sub-districts with incidence rate. Sub-districts with high malaria incidence were all located in the forested mountain range to the south-west. Most of these sub-districts were in Yala (all sub-districts of Than To and Kabang districts).

Similarly, a confidence interval plot of malaria occurrence was used to divide sub-districts into three groups depending on the placement of these intervals completely above, around, or below the overall mean (Fig. 7, left map). This map shows the pattern of malaria occurrence. It indicated that the mountainous area bordering Malaysia was where malaria had a high incidence rate, and it also had a high occurrence there. This map also shows that the majority of sub-districts in Songkhla, Pattani, and Narathiwat provinces, particularly those connected to the Gulf of Thailand's coastal plain, had low to moderate malaria occurrences.

Table 1 summarizes the characteristics of malaria occurrence and incidence rate. Malaria occurrence was highest in 2008 (19.6%) and lowest in 2020 (3.7%), with the incidence rate was highest in 2008 (14.2 cases per 1000 population) and lowest in 2018 (3.3 cases per 1000 population). The highest occurrence was found in both males and females aged 20–29 years, with the incidence rate increasing with age in both genders. The occurrence and incidence rates were highest in Yala province, while the lowest were in Pattani province.

**Table 1:** Occurrence and incidence rate of malaria cases and social-demographic of malaria cases

Determinant	Occurrence (%)	Incidence rate/1000 population
Year		
2008	19.56	14.17
2009	11.94	8.24
2010	12.38	10.11
2011	7.78	4.82
2012	8.90	8.99
2013	13.25	13.62
2014	12.12	7.41
2015	5.81	4.48
2016	13.65	10.77
2017	8.12	5.25
2018	4.48	3.33
2019	5.91	5.55
2020	3.71	6.12
Gender-age group		
Male		
0–9 years	12.16	8.63
10–19 years	15.58	8.89
20–29 years	17.58	8.75
30–39 years	14.07	9.76
40–49 years	11.65	9.29
50–59 years	8.03	11.06
60–69 years	6.25	12.22
70 + years	3.59	12.07
Female		
0–9 years	11.16	7.89
10–19 years	11.43	8.23
20–29 years	11.50	7.72
30–39 years	9.72	8.70
40–49 years	9.16	8.52
50–59 years	7.11	10.21
60–69 years	4.79	11.09
70 + years	3.32	10.67
Province		
Yala	25.89	12.52
Pattani	4.24	4.16
Narathiwat	9.32	7.40
Songkhla	6.75	6.92

**Fig. 8:**
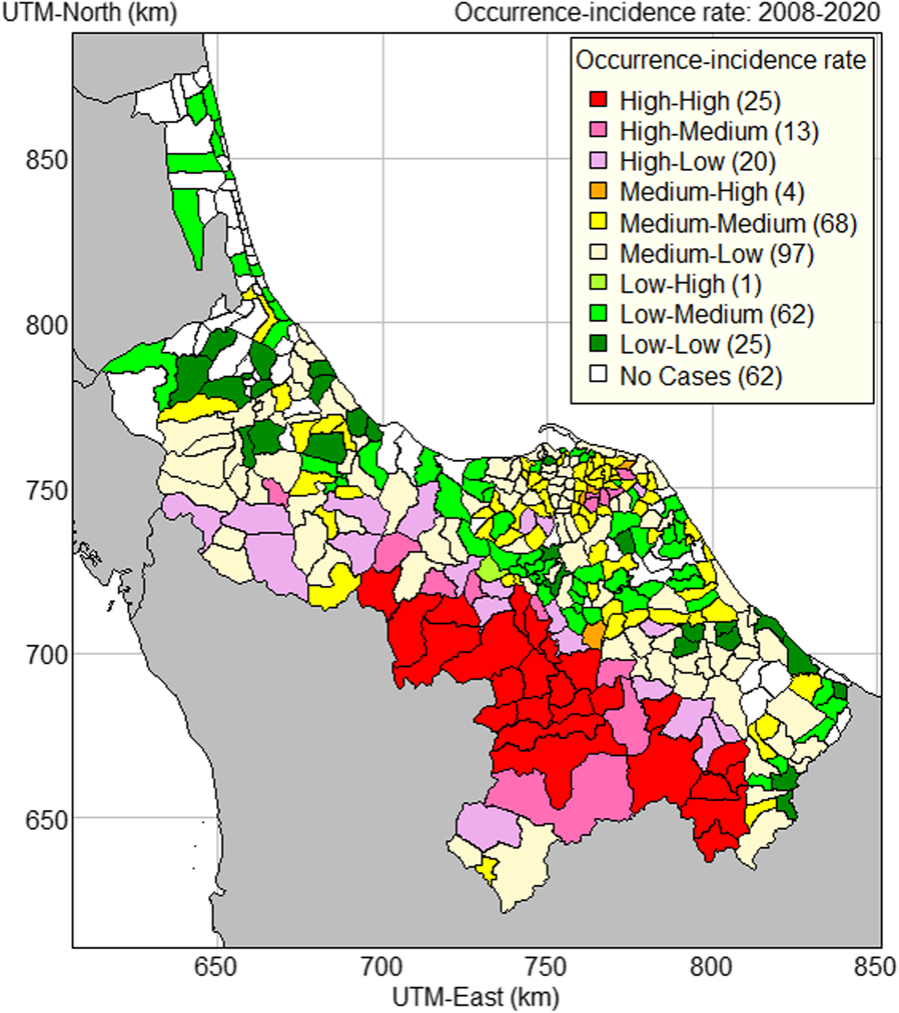
Occurrence-incidence rate map of malaria in 2008–2020 in the four southernmost provinces of Thailand

A thematic map of all combinations of occurrence and incidence rate levels is shown in Fig. 8. The area on this map where malaria occurrence and incidence rate were both high very closely matches the area on the map of the number of cases (Fig. 2, left map) where all the sub-districts reported 25 or more cases over the 13 years.

## Discussion

This work offered an approach to modeling incidence rates in cases when the Poisson and negative binomial distributions failed to fit the data. For such data, a logistic model for disease occurrence and a log-linear regression model for disease incidence rate were fitted independently. This approach provides a much better fit to these data and emphasizes the fact that the incidence rate increased with age for both genders, whereas the occurrence rate peaked at young adult ages and subsequently declined with age for both genders. The occurrence and incidence rates of malaria varied, but both appeared to be declining. High malaria occurrence and incidence rates were found in a cluster of sub-districts in Yala and Narathiwat that bordered Malaysia and had mountainous terrain.

The method of having separate models for occurrence and incidence rate provides a variety of benefits, especially when the predictors show different patterns. The occurrence patterns and incidence rates in this example differ by age. This method can be used to identify areas with high malaria occurrence and incidence rates among susceptible persons, allowing health officials to take preventative actions to reduce the severity of impending epidemics. It can be applied to count distributions in many other fields, including ecology and environmental research, when the data distributions are strongly right-skewed and have a significant proportion of zeros. For instance, researching the environmental factors that are associated to a species' abundance through the study of its ecology. A finer spatial scale analysis of count data with a similar pattern can be done using this technique.

In this study, the occurrence peaked in early adulthood aged 20–29 years and subsequently diminished with age in both genders. These results are in line with the previous study's findings, which showed that Peninsular Malaysia’s malaria cases were highest among people aged 20–29 [25]. Since the majority of the people in this age group are of working age, they generally work in the agricultural and labor-force sectors which spend time outdoors more than other age groups [14, 26]. They have a higher chance to be bitten by a mosquito, especially for those who work in forestry, agriculture, or social services, as well as in plantations or agriculture resulting in a higher occurrence of malaria [27, 28]. Other risks may arise from the improper use of insecticide-treated nets or long-lasting insecticidal nets [29–31]. Defensive strategies or exposure to malaria prevention techniques used in national malaria control initiatives at the household level may have an impact on lowering the occurrence of malaria [12].

In this study, malaria incidence increased with age in both genders, peaked in the 60–69 age group, and then fell in the senior group (over 70 years). These findings contradict previous study conducted in the upper south of Thailand that found a high incidence rate in the 15–44 age group [32]. It should be noted that although these findings suggested that malaria occurred more frequently in younger age groups, older age groups saw a higher proportion of cases if the disease became widespread. This might be because older people tend to live and work mostly in rural areas, which provide favourable environmental conditions for *Anopheles* mosquito breeding grounds, as opposed to younger people, who typically attend school or work in urban areas [9]. In rural areas, malaria control and eradication are more challenging.

Even with the fluctuating occurrence and incidence rates of malaria, there appeared to be a downward tendency. This decrease was attributed to national policies for active management of foci, which included the full adoption of the 1–3-7 surveillance method for persistent active foci [12, 21, 33].

Malaria occurrence and incidence rates were both high in clusters of Yala and Narathiwat provinces’ sub-districts. These all sub-districts, which shares a border with Malaysia, has been identified as a high-risk area for also other mosquito-borne diseases especially Yala province had the highest incidence of malaria in the country in 2016 [9, 16]. The available information in the literature for why this province had a high malaria incidence rate is lacking, however it may be because Yala has a large number of pocket sub-districts spatially related to malaria on the border. The Sankalakhiri mountain range, located on the Thai-Malaysian border, is covered in forest and has a humid climate, making it a preferred habitat for *Anopheles* mosquitoes [9]. Border malaria is complex in terms of both setting and dynamics, as a result of the links between human settlements and transportation activities [18]. In addition, disease control efforts by health personnel visiting the trouble spots were hampered by the unrest situation in the lower southern region [22].

Naturally, mosquito-borne malaria infection occurs as a result of risk behaviors, most notably those associated with improper use of insecticide-treated nets, long-lasting insecticidal nets, and other defensive measures, or those revealed by the household-level implementation of malaria control measures recommended by National Malaria Control Programmes [9, 22]. These major hotspot regions should be investigated in greater detail so that elimination activities can be targeted.

Although this study presents informative findings, it does have limitations. Spatial correlation in malaria incidence among neighbouring sub-districts was not assessed by the proposed model. Further investigation is needed. Moreover, environmental characteristics of the sub-districts, for example, land use and land cover, are not included in the analysis. This aspect seems useful to explore in further studies.

## Conclusions

Malaria incidence rates peaked in older age groups for both genders, while the occurrence peaked in early adulthood for both genders and clusters along the Thai-Malaysian border of Yala and Narathiwat provinces. Malaria prevention and control efforts should be reinforced, with a particular focus on adults and communities living near forest fringes to keep track of progress toward the 2024 target of eradicating malaria. Local border crossings and cross-border migration must be screened. Thailand's Ministry of Public Health and Malaysia's Ministry of Health should cooperate.

## Data Availability

The datasets used and/or analysed during the current study are available from the corresponding author on reasonable request.
